# Risk factors and clinical implications of thyroxine replacement therapy on major adverse cardiovascular events in type 2 diabetes: a retrospective cohort study

**DOI:** 10.3389/fendo.2025.1721865

**Published:** 2026-01-09

**Authors:** Chih-Wei Hsu, Chia-Hung Lin, Pi-Hua Liu, Yi-Hsuan Lin

**Affiliations:** 1 Division of Endocrinology and Metabolism, Department of Internal Medicine, Chang Gung Memorial Hospital, Linkou, Taiwan; 2 Department of Chinese Medicine, College of Medicine, Chang Gung University, Taoyuan, Taiwan; 3 Graduate Institute of Clinical Medical Sciences, Chang Gung University, Taoyuan, Taiwan

**Keywords:** major adverse cardiovascular events, peripheral artery disease, risk factors, thyroxine supplement, type 2 diabetes

## Abstract

**Background:**

Thyroid hormone replacement therapy is widely used to treat hypothyroidism, but there is limited research on its effects in patients with diabetes mellitus (DM). Some studies indicate that this therapy might improve lipid profiles in DM patients, but even with normalized TSH levels from thyroxine replacement, LDL and total cholesterol levels remain higher than in people with normal thyroid function. Additionally, the effect of this therapy on major adverse cardiovascular events (MACE) in DM patients is still uncertain.

**Aim:**

This retrospective study investigated the occurrence of major adverse cardiovascular events (MACE) in participants receiving thyroxine with diabetes and compared the risk factors between the MACE and non-MACE groups.

**Methods:**

We used longitudinal claims data from 2008 to 2017 from the Chang Gung Research Database. Individuals with diabetes who used thyroxine were included. The primary outcome was the occurrence of MACE. The secondary outcomes were the differences between the two groups (MACE vs. no MACE).

**Results:**

After 1:1 group matching by propensity score between MACE and non-MACE groups by sex, age, and interval of using thyroxine, there were 416 patients in each group. Patients with worse renal function (eGFR <45 ml/min/1.73 m^2^), hypertension, history of diabetic microvascular complications, end-stage renal disease (ESRD), coronary heart disease (CHD), heart failure, cerebrovascular accident (CVA), and diabetic foot infection had a higher risk of experiencing MACE. Free T4 had a weak positive correlation with HDL, and TSH had a weak positive correlation with LDL and a negative correlation with HDL (correlation coefficient, *p*-value: 0.131, 0.022; 0.124, 0.016; and -0.157, 0.003, respectively). There were no optimal cutoff points according to the receiver operating characteristic (ROC) curve analysis of the best discrimination point between TSH/free T4/LDL and MACE attack.

**Conclusions:**

In participants receiving thyroxine with diabetes, patients with worse renal function, hypertension, history of diabetic microvascular complications, ESRD, CHD, heart failure, CVA, and diabetic foot infection had a higher risk of experiencing MACE, but peripheral artery disease (PAD) was not a significant risk of MACE.

## 1 Background

Cardiovascular disease (CVD) is a major cause of morbidity and mortality worldwide, and an abnormal lipid profile, characterized by elevated levels of low-density lipoprotein cholesterol (LDL-C) and triglycerides (TG) and low levels of high-density lipoprotein cholesterol (HDL-C), mainly contributes to atherosclerosis and is a well-established risk factor for CVD. Furthermore, there were several other well-known risk factors for CVD, including hypertension, diabetes mellitus (DM), obesity, and cigarette smoking (1). In addition to this, peripheral artery disease (PAD), one of the atherosclerotic diseases, also played an important role in cardiovascular and cerebrovascular ischemic events (2).

The thyroid gland regulates lipid metabolism, blood pressure, vasculature, and angiogenesis (3), and alterations in thyroid function can have a significant impact on lipid profiles (4) by regulating the expression of lipolytic and lipogenic genes (5). Numerous research studies have indicated that variations in thyroid function, such as hypothyroidism and hyperthyroidism, can have a significant impact on lipid metabolism. In individuals with hypothyroidism, LDL-C, TG, and total cholesterol levels are elevated, while HDL-C levels are decreased (6). This condition can potentially increase the risk of atherosclerosis. Furthermore, hypothyroidism has been found to disrupt blood pressure regulation, potentially leading to the development of systolic and diastolic high blood pressure due to increased vascular resistance (7) and arterial stiffness (8). Additionally, hypothyroidism affects the vasculature by causing endothelial dysfunction (9), which is an early stage of atherosclerosis. This has been linked to a decrease in NO availability, further indicating a relationship between hypothyroidism and atherosclerosis (10). In contrast, hyperthyroidism is associated with reduced levels of LDL-C and total cholesterol, with no significant effect on HDL-C levels (11).

Although thyroid hormone replacement therapy is commonly used to treat hypothyroidism, there is a paucity of research on the effects of thyroid hormone replacement therapy in patients with DM. While some studies have suggested that thyroid hormone replacement therapy may improve lipid profiles in patients with DM (12), even after achieving a normal TSH following thyroxine replacement, the LDL-C and total cholesterol levels were still higher than in individuals with normal thyroid function (13). Moreover, the impact of thyroid hormone replacement therapy on major adverse cardiovascular events (MACE) in patients with DM remains unclear.

In this study, we aim to investigate the effects of thyroid hormone replacement therapy on the lipid profile and MACE risk in patients with DM. By elucidating the impact of thyroid hormone replacement therapy on lipid metabolism and CVD risk in this population, we hope to provide valuable insights into the management of dyslipidemia and CVD in patients with DM.

## 2 Materials and methods

### 2.1 Data source

We collected an existing claims dataset to establish a retrospective cohort study from 2008 to 2017 from the Chang Gung Research Database (CGRD), which is a de-identified database of medical records from CGMH, Linkou branch. The CGMH, Linkou branch, founded in 1978, is one of the largest medical institutions in Taiwan. Currently, it has a total number of approximately 3,700 beds, and each year it serves 4 million outpatient visits, 200,000 emergency visits, and 100,000 inpatients. This study was approved by the CGMH Institutional Review Board (IRB).

### 2.2 Codes of interest

We used the International Classification of Diseases, Tenth Revision, Clinical Modification (ICD-10-CM), combined with the Ninth Revision, Clinical Modification (ICD-9-CM), an international medical diagnosis code, based on the timing of the transition, to ascertain the diagnosis of DM, hypothyroidism, atherosclerotic cardiovascular disease, and other underlying diseases of these participants including hypertension (HTN), diabetic microvascular complications, which encompassed diabetic nephropathy, neuropathy, and retinopathy, end-stage renal disease (ESRD), peripheral artery disease (PAD), coronary heart disease (CHD), heart failure, cerebrovascular accident (CVA), and lower extremity amputation (LEA) (**Supplementary Table S1**).

Patients with diabetes mellitus (DM) were identified using the first three digits of the ICD-9 (250) or ICD-10 (E11) codes. Patients were classified as having DM if these codes appeared more than three times among the first three diagnoses in outpatient records or more than once among the first five diagnoses in inpatient records. Among these identified DM patients, those with type 1 DM were excluded based on ICD-9 codes (250.x1 or 250.x3) and ICD-10 code (E10) associated with catastrophic illnesses. The group of people with type 2 diabetes in this study primarily represents the population managed under Taiwan’s National Health Insurance (NHI) system, which provides comprehensive and universal healthcare coverage. Compared to other countries or ethnic backgrounds, the high enrollment rate in the NHI system ensures minimal disparities in healthcare access among different socioeconomic groups, making the dataset representative of the general population with type 2 diabetes in Taiwan.

Furthermore, it is important to note that in Taiwan, type 1 diabetes is classified as a catastrophic illness under the NHI system. This designation allows patients with type 1 diabetes to receive significant reductions in medical expenses. However, obtaining this status requires a rigorous review process to confirm the diagnosis and eligibility for catastrophic illness coverage. As a result, the differentiation between type 1 and type 2 diabetes is carefully validated within the system, ensuring that the study population is accurately categorized and representative of individuals with type 2 diabetes.

Patients were identified as using thyroid medications if their records included the following codes for thyroxine: PMG027M, PMG026M, PMG018M, P2A091M, or P2A093M. Additionally, patients using anti-thyroid drugs were identified based on the following codes: PMG008M, PMG004M, and PMG024M. These anti-thyroid drugs include methimazole, carbimazole, and propylthiouracil.

Patients were prescribed thyroxine due to various forms of hypothyroidism. Primary hypothyroidism was identified using ICD-9 codes 243 or 244.9 and ICD-10 code E03.9. Secondary hypothyroidism was identified using ICD-9 code 244.8 and the same ICD-10 code E03.9. Post-procedure hypothyroidism was identified using ICD-9 codes 244.0, 244.1, 244.2, or 244.3, along with the ICD-10 code E03.9 combined with the procedure codes 0GBG, 0GBH, or 0GBJ. Patients were prescribed thyroxine primarily due to hyperthyroidism with suppression and supplement therapy, which was identified using ICD-9 codes 242.80, 242.81, and 242.9x or ICD-10 codes under the category E05.XX and combined with anti-thyroid drugs and thyroxine usage simultaneously. For those who were receiving thyroxine supplements but did not fall into the aforementioned categories, they were classified into an “other” group.

### 2.3 Outcomes of interest

This study aims to examine the incidence of major adverse cardiovascular events (MACE) in individuals with type 2 diabetes taking thyroxine and to compare the risk factors between those who experienced MACE and those who did not.

MACE is defined as non-fatal myocardial infarction (MI), stroke, or heart failure leading to hospital admission. These events were identified using ICD-9 and ICD-10 codes, with criteria including the top five diagnosis codes for any hospital admission, the top three diagnosis codes appearing more than twice in outpatient visits, or the presence of MACE-related procedural codes or thrombolysis therapy in either inpatient or outpatient settings. The index date was established as the first date meeting the MACE criteria. The aforementioned codes are listed in **Supplementary Table S1**.

### 2.4 Study population

**Figure 1:**
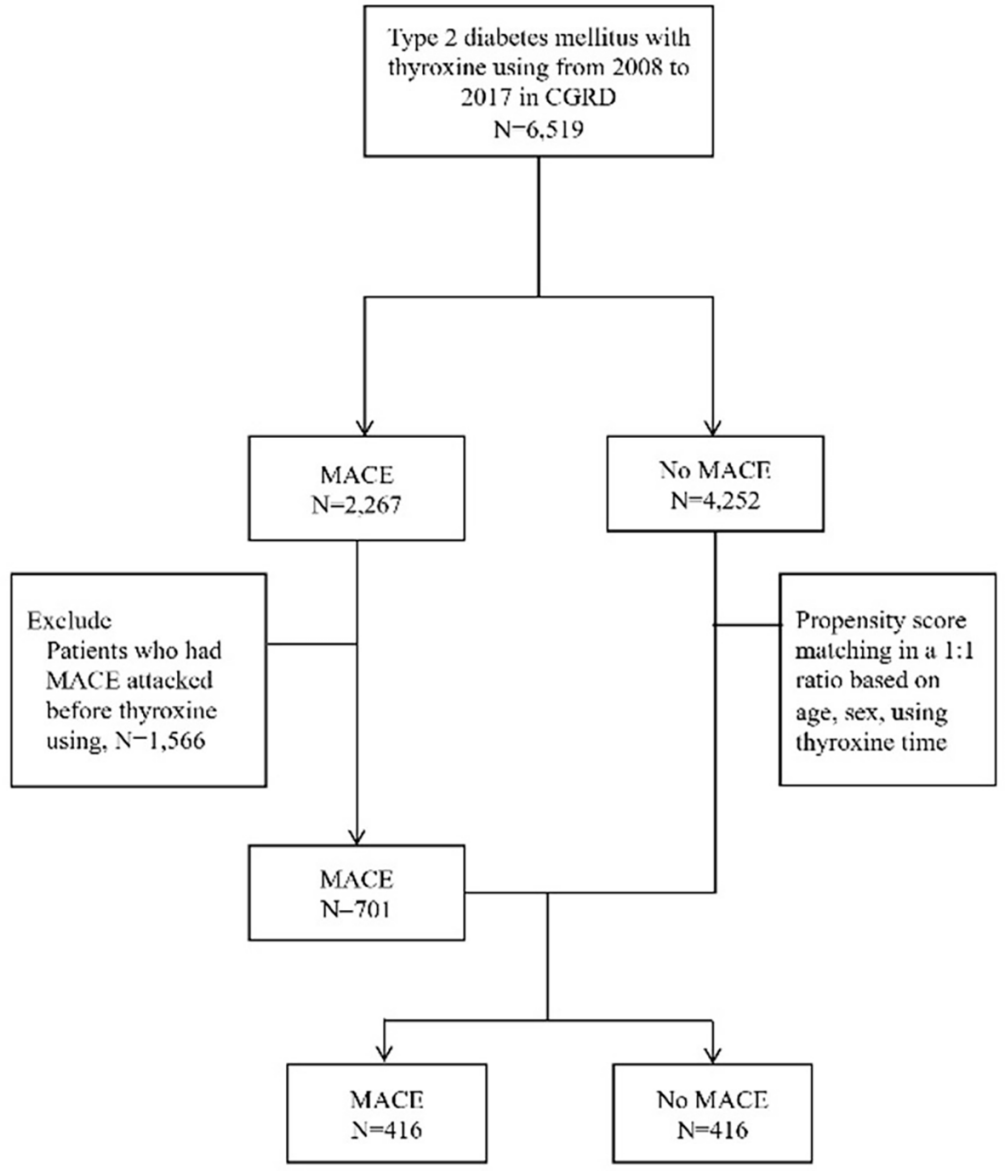
Flowchart of subject recruitment from the CGRD.

From 2008 to 2017, a total of 6,519 individuals with type 2 diabetes who used thyroxine were registered in the CGRD. Among these patients, during the study period, there were 2,267 patients who had MACE and 4,252 patients who did not. We excluded 1,566 patients who had MACE before thyroxine use, and there were 701 patients left. Then, we performed 1:1 propensity-score matching between the MACE and non-MACE groups by sex, age, and interval of using thyroxine. After group matching, we analyzed the MACE and non-MACE groups with 416 patients in each group (**Figure 1**).

These participants received thyroxine for reasons such as primary or secondary hypothyroidism, post-procedure hypothyroidism, hyperthyroidism with suppression and supplement therapy, and other conditions. Biochemical data collected within 1 year before the occurrence of MACE included serum creatinine, HbA1c, eGFR, urine albumin/creatinine ratio (UACR), aspartate aminotransferase (AST), alanine aminotransferase (ALT), uric acid, thyroid-stimulating hormone (TSH), free-T4, T3, thyroid peroxidase antibodies (anti-TPO), anti-thyroid stimulating hormone receptor antibodies (anti-TSH), TG, LDL-C, HDL-C, and thyroglobulin (Tg).

We also analyzed the use of cardiovascular and diabetic medications, including angiotensin-converting enzyme inhibitors/angiotensin receptor blockers (ACEi/ARB), diuretics, statins, ezetimibe, anti-platelet drugs, metformin, sulfonylurea, thiazolidinedione (TZD), acarbose, dipeptidyl peptidase-4 inhibitors (DPP-4i), glucagon-like peptide-1 receptor agonists (GLP-1 RA), and sodium-glucose cotransporter-2 inhibitors (SGLT-2i). These medications were prescribed at least 3 months before a MACE event or at least 3 months before the last follow-up, in conjunction with at least 3 months of thyroxine use in those without MACE.

### 2.5 Data quality checks

We conducted range checks for laboratory values (flagging extreme biologically implausible results), verified the temporal ordering of exposure and outcomes (excluded MACE prior to first LT4 prescription), and required consistent patient identifiers across all tables. Ambiguous duplicate encounters were resolved by deterministic rules (inpatient > emergency > outpatient).

### 2.6 Statistical analysis

Baseline characteristics were compared between groups using independent *t*-tests and analysis of variance (ANOVA) for continuous variables, while categorical variables were assessed using the chi-squared test. Propensity scores were estimated using logistic regression models, followed by the creation of matched pairs to minimize differences in baseline characteristics between the two groups. A propensity score represents the probability of an individual being assigned to a particular treatment group, given their observed baseline characteristics. This method is commonly used in observational studies to reduce potential confounding by balancing covariates across comparison groups. In our analysis, confounding factors such as age, sex, and index date were adjusted for in the logistic regression models used to calculate the propensity scores. By matching participants with similar propensity scores, we aimed to mimic randomization and ensure a more balanced comparison between groups. The association between baseline factors, drug use, and the occurrence of MACE was assessed using univariate and multivariate logistic regression models, with results presented as odds ratios (ORs) and 95% confidence intervals (CIs).

Diagnostic performance was evaluated using the area under the curve (AUC) of the receiver operating characteristic (ROC) curve, with the optimal cutoff point determined by the shortest distance to the point of sensitivity = 1 and 1-specificity = 0.

All statistical analyses were conducted using SAS statistical software version 9.4 (SAS Institute Inc., Cary, NC, USA), and two-sided *p*-values <0.05 were considered statistically significant.

## 3 Results

### 3.1 Demographic characteristics according to the indication for thyroxine use

The demographic characteristics of participants receiving thyroxine, including sex, age, and biochemistry data, are presented in **Table 1**. We separated these participants into four groups according to the causes of hypothyroidism. The patients using thyroxine due to or with secondary hypothyroidism were older (65.48 vs. 63.33 and 61.88 and 63.61 years old, respectively). The proportion of female participants was 95.83% in the group of hyperthyroidism with suppression and supplement therapy, which was higher than that in other groups. Besides that, patients using thyroxine due to primary hypothyroidism or secondary hypothyroidism had higher creatinine and lower eGFR level (1.72 vs. 1.24, 0.96, 1.35 mg/dL, and 63.07 vs. 72.87, 77.07, 71.25 mL/min/1.73 m^2^, respectively) compared to the other three groups. Moreover, the group of primary hypothyroidism or secondary hypothyroidism had a higher proportion of diabetic microvascular complications, ACEi/ARB usage, and diuretics usage.

**Table 1:** Demographic characteristics of participants receiving thyroxine.

Variables	Primary hypothyroidism or secondary hypothyroidism (*n* = 316)	Post-procedure hypothyroidism (*n* = 151)	Hyperthyroidism with suppression and supplement therapy (*n* = 24)	Other (*n* = 341)	*p*-value
Age (year)	65.48 ± 10.66	63.33 ± 10.01^a^	61.88 ± 9.92	63.61 ± 9.99^a^	0.040*
Sex (*n*, %)					0.002*
Female	232 (73.42)	126 (83.44)^a^	23 (95.83)^a^	241 (70.67)^bc^	
Male	84 (26.58)	25 (16.56)^a^	1 (4.17)^a^	100 (29.33)^bc^	
Hypertension (*n*, %)	198 (62.66)	104 (68.87)	16 (66.67)	215 (63.05)	0.570
Diabetic microvascular complications (*n*, %)	135 (42.72)	63 (41.72)	8 (33.33)	107 (31.38)^ab^	0.015*
ESRD (*n*, %)	9 (2.85)	3 (1.99)	0 (0)	6 (1.76)	0.679
PAD (*n*, %)	1 (0.32)	1 (0.66)	0 (0)	1 (0.29)	0.912
CHD	74 (23.42)	33 (21.85)	3 (12.5)	70 (20.53)	0.567
Heart failure	41 (12.97)	19 (12.58)	2 (8.33)	36 (10.56)	0.731
CVA	59 (18.67)	19 (12.58)	5 (20.83)	60 (17.6)	0.391
Diabetic foot infection (*n*, %)	5 (1.58)	5 (3.31)	1 (4.17)	5 (1.47)	0.431
LEA (*n*, %)	1 (0.32)	0 (0)	0 (0)	2 (0.59)	0.769
HbAlc (%, mmol/mol)	7.17 ± 1.66 (213)	7.26 ± 1.2 (100)	6.94 ± 1.29 (20)	7.15 ± 1.61 (216)	0.846
Creatinine (mg/dL)	1.72 ± 2.12 (269)	1.24 ± 1.67 (120)^a^	0.96 ± 0.73 (21)	1.35 ± 1.6 (248)^a^	0.025*
eGFR (mL/min/1.73 m^2^)	63.07 ± 31.96 (269)	72.87 ± 27.47 (120)^a^	77.07 ± 30.03 (21)^a^	71.25 ± 30.18 (248)^a^	0.002*
UACR (mg/g)	365.44 ± 972.63 (59)	388.15 ± 1353.65 (38)	529.02 ± 1254.2 (6)	330.23 ± 1119.87 (64)	0.977
AST (U/L)	39.31 ± 42.58 (172)	29.85 ± 17.72 (60)	43.71 ± 33.56 (14)	31.84 ± 17.56 (152)	0.061
ALT (U/L)	31.84 ± 41.80 (249)	27.23 ± 23.52 (103)	35.58 ± 33.85 (19)	28.98 ± 21.36 (221)	0.502
Uric acid (mg/dL)	6.20 ± 1.98 (119)	6.22 ± 1.97 (45)	7.13 ± 2.57 (9)	6.13 ± 1.59 (110)	0.485
TSH (µIU/mL)	12.53 ± 29.96 (204)	8.27 ± 21.3 (91)	6.07 ± 15.44 (18)	6.35 ± 22.92 (173)	0.116
Free T4 (ng/dL)	1.07 ± 0.39 (178)	1.26 ± 0.40 (74)^ad^	1.38 ± 0.8 (18)^ad^	1.13 ± 0.39 (144)	<0.001*
T3 (ng/dL)	72.73 ± 23.34 (50)	86.30 ± 50.78 (20)	148.16 ± 84.7 (6)^abd^	77.95 ± 36.45 (42)	<0.001*
Anti-TPO Ab (IU/mL)	85.539 ± 177.179 (110)	25.719 ± 64.188 (37)	158.44 ± 242.176 (13)	122.107 ± 277.816 (93)^b^	<0.001*
Anti-TSH Ab (IU/mL)	0.78 ± 0.49 (4)	0	0	4 ± 4.5 (6)	0.200
TG (mg/dL)	153.33 ± 95.78 (208)	162.86 ± 99.81 (100)	151.33 ± 104.92 (18)	153.23 ± 127.85 (217)	0.890
LDL-C (mg/dL)	103.68 ± 38.42 (204)	103.96 ± 35.22 (96)	88.27 ± 38.33 (18)	102.16 ± 33.16 (209)	0.358
HDL-C (mg/dL)	47.64 ± 13.98 (195)	47.34 ± 12.77 (94)	45.38 ± 15.75 (18)	47.87 ± 14.23 (198)	0.905
ACEi/ARB usage (*n*, %)	108 (34.18)	58 (38.41)	8 (33.33)	91 (26.69)^ab^	0.046*
Diuretics usage (*n*, %)	86 (27.22)	27 (17.88)^a^	9 (37.5)^b^	54 (15.84)^ac^	<0.001*
Metformin (*n*, %)	73 (23.1)	44 (29.14)	5 (20.83)	80 (23.46)	0.483
Sulphonylurea/glinide (*n*, %)	90 (28.48)	51 (33.77)	7 (29.17)	84 (24.63)	0.215
TZD (*n*, %)	14 (4.43)	4 (2.65)	1 (4.17)	16 (4.69)	0.766
Acarbose (*n*, %)	28 (8.86)	9 (5.96)	3 (12.5)	28 (8.21)	0.619
DPP-4i (*n*, %)	73 (23.1)	29 (19.21)	5 (20.83)	65 (19.06)	0.599
GLP-1 RA (*n*, %)	5 (1.58)	0 (0)	1 (4.17)	3 (0.88)	0.199
SGLT2i (*n*, %)	5 (1.58)	2 (1.32)	0 (0)	6 (1.76)	0.914
Anti-platelet (*n*, %)	81 (25.63)	39 (25.83)	2 (8.33)	77 (22.58)	0.226
Statin (*n*, %)	105 (33.23)	47 (31.13)	8 (33.33)	107 (31.38)	0.951
Ezetimibe (*n*, %)	20 (6.33)	8 (5.3)	3 (12.5)	18 (5.28)	0.507

HTN, hypertension; ESRD, end-stage renal disease; PAD, peripheral artery disease; CHD, coronary heart disease; CVA, cerebrovascular accident; LEA, lower extremity amputation; HbA1c, glycated hemoglobin; eGFR, estimated glomerular filtration rate; UACR, urine albumin to creatinine ratio; AST, aspartate aminotransferase; ALT, alanine aminotransferase; TSH, thyroid-stimulating hormone; anti-TPO Ab, antithyroid peroxidase antibody; TG, triglyceride; LDL-C, low-density lipoprotein cholesterol; HDL-C, high-density lipoprotein cholesterol; ACEi, angiotensin-converting enzyme inhibitor; ARB, angiotensin receptor blockers; TZD, thiazolidinedione; DPP-4i, dipeptidyl peptidase-4 inhibitor; GLP-1 RA, glucagon-like peptide-1 receptor agonist; SGLT-2i, sodium-glucose co-transporter 2 inhibitor.

*p-value < 0.05.

a Significant difference with primary hypothyroidism or secondary hypothyroidism group.

b Significant difference with post-procedure hypothyroidism group.

c Significant difference with hyperthyroidism with suppression and supplement therapy group.

d Significant difference with other groups.

### 3.2 Correlation between thyroid function, lipid profile, and HbA1c

The relationship between thyroid function, lipid profile, and HbA1c was analyzed by using Pearson correlation coefficient (**Table 2**). Free T4 had a weak positive correlation with HDL (correlation coefficient, *p*-value: 0.131 and 0.022, respectively), and TSH had a weak positive correlation with LDL and negative correlation with HDL (correlation coefficient, *p*-value: 0.124, 0.016; -0.157, 0.003, respectively).

**Table 2:** Correlation between thyroid function, lipid profile, and HbA1c.

Variables	Free T4		T3		TSH		Anti-TPO Ab		Anti-TSH Ab	
	Correlation coefficient	*p*-value	Correlation coefficient	*p*-value	Correlation coefficient	*p*-value	Correlation coefficient	*p*-value	Correlation coefficient	*p*-value
LDL	-0.006	0.916	0.019	0.868	0.124	0.016^a^	-0.032	0.641	-0.265	0.491
HDL	0.131	0.022^a^	0.043	0.701	-0.157	0.003^a^	0.159	0.023^a^	-0.144	0.711
HbA1c	-0.040	0.467	-0.050	0.644	-0.053	0.294	-0.026	0.706	0.495	0.175

a *p*-value <0.05.

TSH, thyroid-stimulating hormone; Anti-TPO Ab, antithyroid peroxidase antibody; LDL-C, low-density lipoprotein cholesterol; HDL-C, high-density lipoprotein cholesterol; HbA1c, glycated hemoglobin.

### 3.3 Subgroup analyses of MACE risk factors

**Figure 2:**
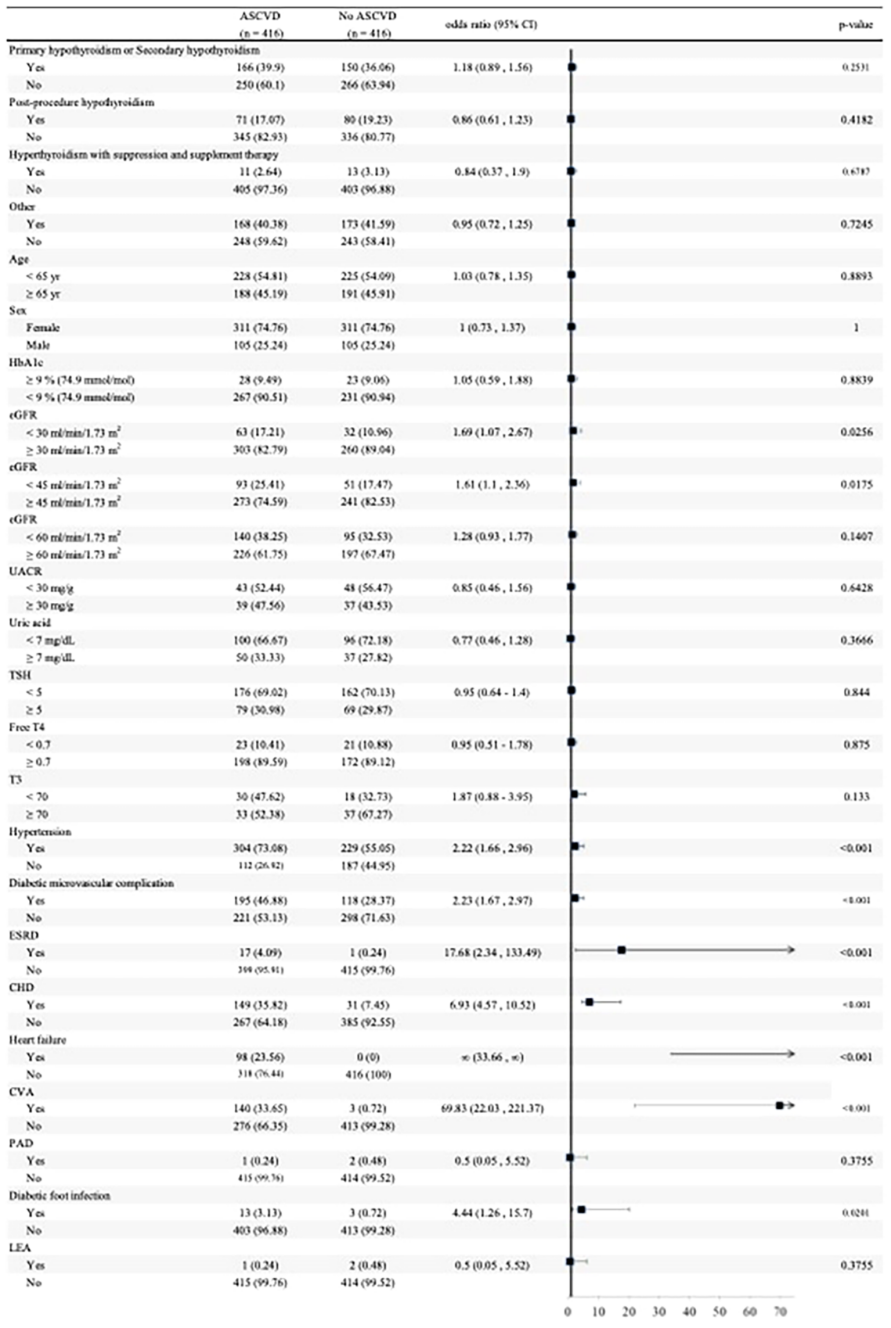
Odds ratios with 95% confidence intervals for MACE in patients with type 2 diabetes mellitus receiving thyroxine, derived from logistic regression models. Values are shown as the number of patients and percentage in each subgroup. MACE, major adverse cardiovascular events; CI, confidence interval; HbA1c, glycated hemoglobin; eGFR, estimated glomerular filtration rate; UACR, urine albumin to creatinine ratio; TSH, thyroid-stimulating hormone; ESRD, end-stage renal disease; CHD, coronary heart disease; CVA, cerebrovascular accident; PAD, peripheral artery disease; LEA, lower extremity amputation.

The subgroup analyses of MACE risk factors are summarized in **Figure 2**. Patients with worse renal function (eGFR <45 ml/min/1.73 m^2^), hypertension, history of diabetic microvascular complications, ESRD, CHD, heart failure, CVA, and diabetic foot infection had a higher risk of experiencing MACE. On the other hand, indications for LT4 prescription, PAD, and LEA were not risk factors for experiencing MACE among participants receiving thyroxine. Furthermore, we also used the TSH level 5 µIU/mL, free T4 level 0.7 ng/dL, and T3 level 70 ng/dL as cut point because these values were the closest to average, but there was no significant finding of a different occurrence rate of MACE.

Moreover, we used univariate and multivariate logistic regression to analyze the risk factors on MACE (**Table 3**).

**Table 3:** Predictors of MACE in participants with type 2 diabetes receiving thyroxine therapy, assessed by logistic regression.

Variables	Univariate		Multivariate model 1		Multivariate model 2		Multivariate model 3		Multivariate model 4	
	Odds ratio (95% CI)	*P*-value	Odds ratio (95% CI)	*P*-value	Odds ratio (95% CI)	*P*-value	Odds ratio (95% CI)	*P*-value	Odds ratio (95% CI)	*P*-value
Age										
<65 years	1.03 (0.78–1.35)	0.8893					1.59 (0.94–2.72)	0.087	1.57 (0.92–2.68)	0.0974
≥65 years										
Sex										
Female	1 (0.73–1.37)	1					1.08 (0.59–1.95)	0.8106	1.12 (0.62–2.02)	0.6973
Male										
eGFR										
<30 mL/min/1.73 m^2^	1.69 (1.07–2.67)	0.0256*	1.44 (0.65–3.18)	0.3669			1.55 (0.68–3.51)	0.2961		
≥30 mL/min/1.73 m^2^										
eGFR										
<45 mL/min/1.73 m^2^	1.61 (1.1–2.36)	0.0175*			1.1 (0.54–2.21)	0.7957			1.19 (0.58–2.43)	0.6363
≥45 mL/min/1.73 m^2^										
TSH										
<5	0.95 (0.64–1.4)	0.8437	0.55 (0.22–1.35)	0.1932	0.64 (0.36–1.14)	0.1297	0.65 (0.36–1.15)	0.1394	0.64 (0.36–1.13)	0.1224
≥5										
Free T4										
<0.7	0.95 (0.51–1.78)	0.8747	0.66 (0.37–1.16)	0.1487	0.55 (0.22–1.34)	0.1884	0.58 (0.23–1.44)	0.2397	0.58 (0.23–1.44)	0.2404
≥0.7										
Hypertension										
Yes	2.22 (1.66–2.96)	<0.001*	2.2 (1.21–3.98)	0.0097*	2.27 (1.26–4.11)	0.0066*	2.38 (1.29–4.37)	0.0053*	2.44 (1.33–4.47)	0.0038*
No										
Diabetic microvascular complication										
Yes	2.23 (1.67–2.97)	<0.001*	0.91 (0.51–1.61)	0.7458	0.96 (0.54–1.72)	0.895	0.92 (0.51–1.63)	0.765	0.96 (0.53–1.73)	0.8932
No										
ESRD										
Yes	17.68 (2.34–133.49)	<0.001*	5.42 (0.50–58.22)	0.1631	6.61 (0.63–69.4)	0.1155	4.54 (0.43–47.98)	0.2084	5.53 (0.54–56.96)	0.1504
No										
CHD										
Yes	6.93 (4.57–10.52)	<0.001*	12.58 (6.00–26.40)	<0.001*	12.49 (5.95–26.21)	<0.001*	12.31 (5.86–25.87)	<0.001*	12.26 (5.83–25.78)	<0.001*
No										
CVA										
Yes	69.83 (22.03–221.37)	<0.001*	44.05 (13.06–148.56)	<0.001*	43.69 (12.94–147.53)	<0.001*	45.86 (13.52–155.59)	<0.001*	45.76 (13.46–155.6)	<0.001*
No										
Diabetic foot infection										
Yes	4.44 (1.26–15.7)	0.0201*	2.63 (0.6–11.44)	0.1984	2.43 (0.56–10.61)	0.2387	2.91 (0.65–13.00)	0.1617	2.6 (0.58–11.64)	0.2111
No										

eGFR, estimated glomerular filtration rate; TSH, thyroid-stimulating hormone; ESRD, end-stage renal disease; CHD, coronary heart disease; CVA, cerebrovascular accident.

*p-value <0.05.

### 3.4 Receiver operating characteristic curve analysis of the best discrimination point between TSH/free T4/LDL and MACE

**Figure 3:**
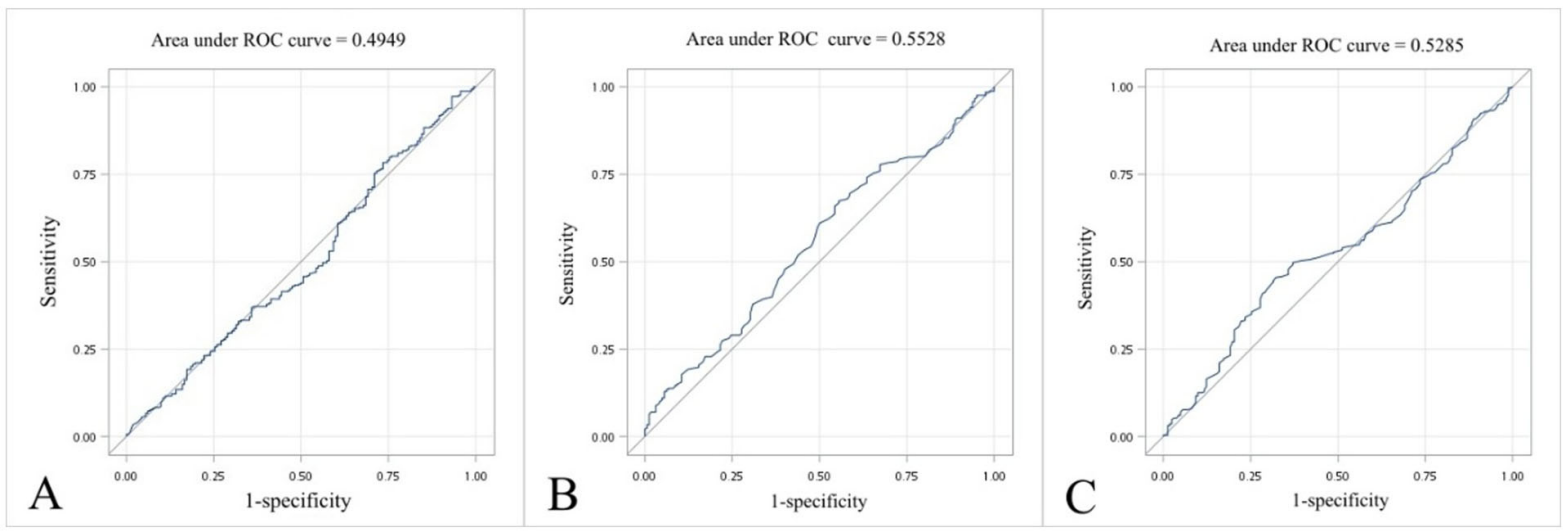
ROC curve of MACE by **(A)** TSH level, **(B)** free T4 level, and **(C)** LDL level. ROC, receiver operating characteristic curve; MACE, major adverse cardiovascular events; TSH, thyroid-stimulating hormone; LDL-C, low-density lipoprotein cholesterol.

To explore the best discrimination point of thyroid function for MACE attack, we tried to analyze the best point by ROC curve, representing the largest sum of sensitivity and specificity. **Figure 3** presents the ROC curves for three predictive models based on different biochemical parameters: (A) TSH level, (B) free T4 level, and (C) LDL level. The area under the curve (AUC) values were 0.4949, 0.5528, and 0.5285, respectively. These values are close to 0.5, indicating only poor discrimination, and we were therefore unable to identify clinically useful cut-off values for TSH, free T4, or LDL in predicting MACE in this cohort.

## 4 Discussion

There were four main findings in our study. First, we did not observe a relationship between T4 or TSH levels and MACE. Second, in the Pearson correlation analysis, free T4 showed a weak positive correlation with HDL, whereas TSH showed a weak positive correlation with LDL and a negative correlation with HDL. Third, impaired renal function, hypertension, a history of diabetic microvascular complications, ESRD, CHD, heart failure, CVA, and diabetic foot infection were all associated with a higher risk of MACE, whereas PAD and LEA were not. Fourth, the ROC curve analysis did not identify an optimal cutoff point of thyroid function for predicting MACE. Our study confirmed that traditional cardiovascular risk factors remain strongly predictive of MACE, independent of thyroid status. We explored whether these factors retained predictive value among patients on LT4 and whether LT4 may modify the risk. While LT4 therapy does not eliminate established risks, it may interact with metabolic and vascular pathways influencing cardiovascular outcomes. Mechanistically, LT4 can improve endothelial function, lipid metabolism, and arterial stiffness (14, 15), but excessive dosing or suboptimal control may increase heart rate, myocardial oxygen demand, or arrhythmogenic risk (16). Therefore, these associations likely reflect a complex interplay rather than causality, which should be interpreted cautiously (15, 16). We discuss our results in comparison with previous studies in the following sections.

Whether levothyroxine therapy itself reduces or increases the risk of MACE in diabetics is an intriguing question, and current evidence is mixed. According to previous studies, thyroid function significantly affects lipid metabolism. Within the reference TSH level, there was a significant positive correlation between TSH level and total cholesterol, LDL-C, non-HDL-C, and TG and a negative correlation with HDL-C. Wang JJ et al. assessed the causal association between thyroid function and lipid metabolism via a genetic analysis, and the results demonstrated that increased TSH levels were significantly associated with higher total cholesterol (TC) and LDL levels, and the FT3/FT4 ratio was significantly associated with TC and LDL levels (17). Jung et al. analyzed the association between thyroid function and lipid profiles, apolipoproteins, and HDL function. The TC, TG, LDL-C, and apoB levels and the apoA-I/II ratio were significantly increased in the overt hypothyroid state and recovered to baseline values with levothyroxine replacement (18). Thyroid hormone influences lipid metabolism in many ways. T3 can mediate gene activation to control lipid metabolism, increase bile acid flow, furtherly enhance serum cholesterol uptake by the liver and regular thermogenesis, and reduce body weight by stimulating brown adipose tissue activity (4, 19). In type 2 diabetes patients, higher TSH and lower T3 and T4 level were noted compared with the non-diabetic control group by a case–control study. Meanwhile, higher serum TC, LDL-C, and TG levels were also seen in type 2 diabetes, and it was obvious that a significant positive correlation between TSH and TC, LDL-C, and TG and negative correlation between T3/T4 and TC, LDL-C, and TG were also found (20). Another retrospective study also disclosed that the TSH level was higher in patients with diabetes than those without diabetes (21). A 2020 meta-analysis specifically examining diabetic patients found no significant association between hypothyroidism and MACE or cardiovascular mortality​, implying that thyroid status (treated or untreated) did not markedly alter the event risk in that pooled analysis (22). Moreover, dysthyroid states also affect the heart, not only the rhythm but also the structure, which furtherly increase mortality and the risk of MACE (23). Although the results of correlation between thyroid function and lipid profile and MACE by previous studies were consistent with those of the current study, there were no previous studies focusing on thyroid level after thyroxine supplementation in type 2 diabetes patients.

Based on real-world data, it seems that treating patients with subclinical hypothyroidism using thyroxine does not provide significant benefits for all-cause mortality and MACE (24). Additionally, in patients with hyperthyroidism, the risk of MACE and heart failure increases (25). Therefore, we further analyzed whether there is a controlled threshold for thyroid hormone concentration that can achieve the lowest incidence of MACE.

In our study, the AUCs for TSH and free T4 were close to 0.5, so no reliable cut-off values for these markers in relation to MACE could be identified. There was a population-based prospective cohort study executed in Finland by Langen et al. which presented that compared with TSH within the reference range, a high TSH level was related to a greater risk of total mortality and sudden cardiac death, whereas a low TSH level was not associated with MACE. The TSH level did not have a linear relation with any of the cardiac outcomes and showed a U-shaped association with total mortality (26). Although the diabetic population was approximately 4.7% in the study, there was no further subgroup analysis.

In this study, in diabetic patients receiving levothyroxine therapy, the presence of PAD did not significantly increase the risk of MACE, contrary to the usual expectation that diabetes combined with PAD markedly elevates cardiovascular risk (27). This non-significant finding should be interpreted cautiously, as the relatively low prevalence of PAD in our cohort may have limited the statistical power to detect an association with MACE. Therefore, the absence of a statistically significant relationship may reflect sample size limitations rather than a true lack of association. According to previous studies, levothyroxine therapy has been associated with improved endothelial function in hypothyroid individuals through several mechanisms, including enhanced nitric oxide (NO) production and reduced endothelial adhesion molecule expression, alongside anti-inflammatory effects (28–31). These thyroid-hormone-mediated benefits generally promote vasodilation and a healthier vascular endothelium. However, in patients with PAD, the underlying disease state is characterized by chronic inflammation, endothelial cell dysfunction, arterial remodeling, and increased vascular stiffness—a pathophysiological milieu that can blunt or outweigh the protective effects of thyroid hormone (32, 33)—for instance, PAD is associated with elevated levels of pro-inflammatory cytokines (e.g., IL-6, TNF-α) and endothelial adhesion molecules, reflecting ongoing vascular inflammation and damage that limit NO bioavailability and normal endothelial responsiveness. The findings of our study may therefore reflect an interplay of these opposing mechanisms: any potential vasoprotective effect of thyroxine is counteracted by the advanced atherosclerotic and inflammatory processes in PAD, such that PAD did not confer a significantly higher MACE risk in the thyroxine-treated diabetic patients (28). This suggests that thyroxine’s protective influence, while biologically plausible, may not fully manifest in the presence of severe vascular pathology.

We also acknowledge that defining LT4 use as ≥3 months introduces variability in treatment duration. Therefore, in the propensity score matching, we further matched the interval of LT4 use to partially account for treatment duration. Longer therapy may allow more stable euthyroid status, improved lipid metabolism, and better endothelial function, whereas shorter or inconsistent use may attenuate the benefits. Future studies with longitudinal thyroid function and medication data are warranted to clarify dose- and duration-dependent effects (14, 34).

To test whether cardiovascular risk varies by the indication for LT4, we performed a prespecified subgroup analysis stratifying users into primary/secondary hypothyroidism, post-procedure hypothyroidism, TSH suppression therapy, and other. In the propensity-matched cohort, none of the indications showed an independent association with MACE. These neutral findings contrast with recent cohorts limited to subclinical hypothyroidism, which reported modest MACE risk reductions with LT4 (16).

Residual misclassification may also attenuate differences because our database records the start date of LT4 but not the subsequent dose titrations or patient adherence. Larger, biochemically phenotyped studies with explicit indication coding and on-treatment TSH are needed to clarify whether MACE risk truly differs by LT4 indication.

This study had some limitations. First, this was a nonrandomized, retrospective, observational study; therefore, selection bias was possible despite comprehensive propensity score matching and our setting the index date as MACE attack. Some patients might not be included if the clinical physicians missed to put the codes on the diagnostic system. Second, biochemical results were incomplete in some patients, and there were missed data. Several biochemical variables, such as serum T3 and UACR, had smaller available sample sizes, which may have reduced the statistical power for certain analyses. Missing data were handled using a complete case approach, and no imputation was performed. Therefore, the results involving these variables should be interpreted with caution due to potential information loss. Third, the sample size was relatively small. Fourth, while prescription timing was available, subsequent dose adjustments and individual adherence to LT4 could not be reliably captured in our dataset, which may lead to exposure misclassification through TSH variability. Fifth, due to database limitations, we could not include important confounders such as BMI and smoking. Variables such as BMI, smoking, and diabetes duration are important confounders for MACE risk. Unfortunately, these parameters were not consistently available in the database and could not be adjusted for in our multivariate models. Nevertheless, we incorporated major cardiometabolic variables such as renal function, lipid profile, anti-hypertensive drug use, and comorbidities to partially account for cardiovascular risk differences. Given the retrospective observational design, the findings of this study should be interpreted as associative rather than causal. Causality cannot be inferred because of potential residual confounding and the absence of temporal verification.

In the future, we can design a prospective study to collect longer follow-up results for these groups of diabetes patients.

Conclusively, in individuals with type 2 diabetes receiving thyroxine therapy, no strong association was observed between T4 or TSH levels and the occurrence of MACE. Conversely, patients with impaired renal function, hypertension, a history of diabetic microvascular complications, ESRD, CHD, heart failure, CVA, or diabetic foot infections demonstrated a significantly higher risk of experiencing MACE. Notably, PAD did not emerge as a significant risk factor for MACE in this cohort.

## Data Availability

The original contributions presented in the study are included in the article/**Supplementary Material**. Further inquiries can be directed to the corresponding author.

## Supplementary material

Supplementary Table 1 Definition and codes of diseases and medicines.

## References

[1] Leritz EC McGlinchey RE Kellison I Rudolph JL Milberg WP . Cardiovascular disease risk factors and cognition in the elderly. Curr Cardiovasc Risk Rep. (2011) 5:407–12. doi: 10.1007/s12170-011-0189-x, PMID: 22199992 PMC3245189

[2] Mejias ELP Faxas SM Taveras NT Talpur AS Kumar J Khalid M . Peripheral artery disease as a risk factor for myocardial infarction. Cureus. (2021) 13:e15655. doi: 10.7759/cureus.15655, PMID: 34277248 PMC8280959

[3] Ichiki T . Thyroid hormone and vascular remodeling. J Atheroscl thrombosis. (2016) 23:266–75. doi: 10.5551/jat.32755, PMID: 26558400

[4] Rizos C Elisaf M Liberopoulos E . Effects of thyroid dysfunction on lipid profile. Open Cardiovasc Med J. (2011) 5:76. doi: 10.2174/1874192401105010076, PMID: 21660244 PMC3109527

[5] Mullur R Liu Y-Y Brent GA . Thyroid hormone regulation of metabolism. Physiol Rev. (2014) 94:355–82. doi: 10.1152/physrev.00030.2013, PMID: 24692351 PMC4044302

[6] Duntas LH Brenta G . The effect of thyroid disorders on lipid levels and metabolism. Med Clinics. (2012) 96:269–81. doi: 10.1016/j.mcna.2012.01.012, PMID: 22443975

[7] Graettinger JS Muenster JJ Checchia CS Grissom RL Campbell JA . A correlation of clinical and hemodynamic studies in patients with hypothyroidism. J Clin Invest. (1958) 37:502–10. doi: 10.1172/JCI103631, PMID: 13539188 PMC293114

[8] Obuobie K Smith J Evans L John R Davies J Lazarus JH . Increased central arterial stiffness in hypothyroidism. J Clin Endocrinol Metab. (2002) 87:4662–6. doi: 10.1210/jc.2002-020493, PMID: 12364455

[9] Lekakis J Papamichael C Alevizaki M Piperingos G Marafelia P Mantzos J . Flow-mediated, endothelium-dependent vasodilatation is impaired in subjects with hypothyroidism, borderline hypothyroidism, and high-normal serum thyrotropin (TSH) values. Thyroid. (1997) 7:411–4. doi: 10.1089/thy.1997.7.411, PMID: 9226212

[10] Taddei S Caraccio N Virdis A Dardano A Versari D Ghiadoni L . Impaired endothelium-dependent vasodilatation in subclinical hypothyroidism: beneficial effect of levothyroxine therapy. J Clin Endocrinol Metab. (2003) 88:3731–7. doi: 10.1210/jc.2003-030039, PMID: 12915662

[11] Biondi B Cooper DS . The clinical significance of subclinical thyroid dysfunction. Endocrine Rev. (2008) 29:76–131. doi: 10.1210/er.2006-0043, PMID: 17991805

[12] Monzani F Caraccio N Kozakowa M Dardano A Vittone F Virdis A . Effect of levothyroxine replacement on lipid profile and intima-media thickness in subclinical hypothyroidism: a double-blind, placebo-controlled study. J Clin Endocrinol Metab. (2004) 89:2099–106. doi: 10.1210/jc.2003-031669, PMID: 15126526

[13] McAninch EA Rajan KB Miller CH Bianco AC . Systemic thyroid hormone status during levothyroxine therapy in hypothyroidism: A systematic review and meta-analysis. J Clin Endocrinol Metab. (2018) 103:4533–42. doi: 10.1210/jc.2018-01361, PMID: 30124904 PMC6226605

[14] Liu L Zhang H Chen X Liu Z Zhao H Ma S . Effect of levothyroxine on major adverse cardiovascular events in patients with hypothyroidism and cardiovascular disease. Front Endocrinol (Lausanne). (2025) 16:1640086. doi: 10.3389/fendo.2025.1640086, PMID: 40933375 PMC12417138

[15] Wang P Zhang W Liu H . Research status of subclinical hypothyroidism promoting the development and progression of cardiovascular diseases. Front Cardiovasc Med. (2025) 12:1527271. doi: 10.3389/fcvm.2025.1527271, PMID: 40255342 PMC12006070

[16] Holley M Razvi S Maxwell I Dew R Wilkes S . Assessing the cardiovascular effects of levothyroxine use in an ageing United Kingdom population (ACEL-UK): cohort study. J Clin Endocrinol Metab. (2025) 110:e4137–43. doi: 10.1210/clinem/dgaf208, PMID: 40179252 PMC12623041

[17] Wang J-J Zhuang Z-H Shao C-L Yu C-Q Wang W-Y Zhang K . Assessment of causal association between thyroid function and lipid metabolism: a Mendelian randomization study. Chin Med J. (2021) 134:1064–9. doi: 10.1097/CM9.0000000000001505, PMID: 33942801 PMC8116035

[18] Jung KY Ahn HY Han SK Park YJ Cho BY Moon MK . Association between thyroid function and lipid profiles, apolipoproteins, and high-density lipoprotein function. J Clin lipidology. (2017) 11:1347–53. doi: 10.1016/j.jacl.2017.08.015, PMID: 28958565

[19] Duntas LH Brenta G . A renewed focus on the association between thyroid hormones and lipid metabolism. Front endocrinology. (2018) 9:511. doi: 10.3389/fendo.2018.00511, PMID: 30233497 PMC6129606

[20] Pangajam P . Association between thyroid hormones & lipid profile in type 2 diabetes mellitus patients-A case control study in tertiary care hospital. Int J Clin Biochem Res. (2023) 8:25–8. doi: 10.18231/j.ijcbr.2021.006

[21] Saha HR Khan H Sarkar BC Khan SA Sana NK Sugawara A . A comparative study of thyroid hormone and lipid status of patient with and without diabetes in adults. Open J Endocrine Metab Diseases. (2013) 3:113–9. doi: 10.4236/ojemd.2013.32017

[22] Faber J Selmer C . Cardiovascular disease and thyroid function. Front Horm Res. (2014) 43:45–56. doi: 10.1159/000360558, PMID: 24943297

[23] Andersen MN Olsen A-MS Madsen JC Kristensen SL Faber J Torp-Pedersen C . Long-term outcome in levothyroxine treated patients with subclinical hypothyroidism and concomitant heart disease. J Of Clin Endocrinol Metab. (2016) 101:4170–7. doi: 10.1210/jc.2016-2226, PMID: 27571183

[24] Selmer C Olesen JB Hansen ML von Kappelgaard LM Madsen JC Hansen PR . Subclinical and overt thyroid dysfunction and risk of all-cause mortality and cardiovascular events: a large population study. J Clin Endocrinol Metab. (2014) 99:2372–82. doi: 10.1210/jc.2013-4184, PMID: 24654753

[25] Langen VL Niiranen TJ Puukka P Lehtonen AO Hernesniemi JA Sundvall J . Thyroid-stimulating hormone and risk of sudden cardiac death, total mortality and cardiovascular morbidity. Clin endocrinology. (2018) 88:105–13. doi: 10.1111/cen.13472, PMID: 28862752

[26] Sprenger L Mader A Larcher B Mächler M Vonbank A Zanolin-Purin D . Type 2 diabetes and the risk of cardiovascular events in peripheral artery disease versus coronary artery disease. BMJ Open Diabetes Res Care. (2021) 9:e002407. doi: 10.1136/bmjdrc-2021-002407, PMID: 34782334 PMC8593703

[27] Razvi S Jabbar A Pingitore A Danzi S Biondi B Klein I . Thyroid hormones and cardiovascular function and diseases. J Am Coll Cardiol. (2018) 71:1781–96. doi: 10.1016/j.jacc.2018.02.045, PMID: 29673469

[28] Marchiori RC Pereira LA Naujorks AA Rovaris DL Meinerz DF Duarte MM . Improvement of blood inflammatory marker levels in patients with hypothyroidism under levothyroxine treatment. BMC Endocrine Disord. (2015) 15:1–9. doi: 10.1186/s12902-015-0032-3, PMID: 26100072 PMC4476077

[29] Patrizio A Ferrari SM Elia G Ragusa F Balestri E Botrini C . Hypothyroidism and metabolic cardiovascular disease. Front Endocrinol (Lausanne). (2024) 15:1408684. doi: 10.3389/fendo.2024.1408684, PMID: 38887272 PMC11180764

[30] Zúñiga D Balasubramanian S Mehmood KT Al-Baldawi S Zúñiga Salazar G . Hypothyroidism and cardiovascular disease: A review. Cureus. (2024) 16:e52512. doi: 10.7759/cureus.52512, PMID: 38370998 PMC10874251

[31] Signorelli SS Marino E Scuto S . Inflammation and peripheral arterial disease. J. (2019) 2:142–51. doi: 10.3390/j2020012

[32] Triggiani V Cittadini A Lisco G . Effect of levothyroxine replacement therapy in patients with subclinical hypothyroidism and chronic heart failure: A systematic review. Front Endocrinol (Lausanne). (2022) 13:1013641. doi: 10.3389/fendo.2022.1013641, PMID: 36457560 PMC9706201

[33] Holley M Razvi S Farooq MS Dew R Maxwell I Wilkes S . Cardiovascular and bone health outcomes in older people with subclinical hypothyroidism treated with levothyroxine: a systematic review and meta-analysis. Syst Rev. (2024) 13:123. doi: 10.1186/s13643-024-02548-7, PMID: 38720372 PMC11077844

[34] Yu OHY Filliter C Filion KB Platt RW Grad R Renoux C . Levothyroxine treatment of subclinical hypothyroidism and the risk of adverse cardiovascular events. Thyroid. (2024) 34:1214–24. doi: 10.1089/thy.2024.0227, PMID: 39104265

