# Supplementary material for: Risk factors and clinical implications of thyroxine replacement therapy on major adverse cardiovascular events in type 2 diabetes: a retrospective cohort study
Source: Front Endocrinol (Lausanne). 2026 Jan 9;16:1721865. doi: 10.3389/fendo.2025.1721865 (PMC12827115; doi:10.3389/fendo.2025.1721865)
Supplement: Supplementary Table 1 — Definition and codes of diseases and medicines. [file Table1.docx]

Supplemental Table S1: Definition and codes of disease and medicine

| **Disease and medicine** | Code and definition |
| --- | --- |
| **Type 1 diabetes mellitus** | ICD-9 Registry for catastrophic illness patients disease code as 250.x1 or 250.x3  ICD-10 Registry for catastrophic illness patients’ disease code as E10 |
| **Type 2 diabetes mellitus** | The below definition excludes Type 1 diabetes  ICD-9 disease code 250 (the first three numbers) and emerge more than three times and in the first three diagnosis in outpatient clinics diagnostic code; or emerge more than once in the first five diagnosis in admission diagnostic code.  ICD-10 disease code as E11 (the first three words) and emerge more than three times and in the first three diagnosis in outpatient clinics diagnostic code; or emerge more than once in the first five diagnosis in admission diagnostic code. |
| Primary hypothyroidism: | ICD-9 disease code 243 or 244.9  ICD-10 disease code E03.9 |
| Secondary hypothyroidism | ICD-9 disease code 244.8  ICD-10 disease code E03.9 |
| Post-procedure hypothyroidism | ICD-9 disease code 244.0, 244.1, 244.2, 244.3  ICD-10 disease code E03.9 combined with procedure codes of 0GBG or 0GBH or 0GBJ |
| Hyperthyroidism | ICD-9 disease code 242.80, 242.81, 242.9x  ICD-10 disease code E05.XX |
| **Below comorbidities (except ESRD) were identified using ICD-9 and ICD-10 codes based on the top five coding for any admission or on the top three coding for more than twice outpatient visits** | |
| **Diabetic Peripheral artery disease (PAD)** | ICD-9 code:   1. 250.7X 2. 250.xx add on one of below:   440.2X, 440.3X, 440.4, 443.81, 443.89, 443.9, 444.22, 445.02, 785.4  ICD-10 code:  1. E0851, E0859, E0951, E0959, E1151, E1159, E1351, E1359  2. E11 add on one of below:  I70201, I70202, I70203, I70208, I70209, I70211, I70212, I70213, I70218, I70219, I70221, I70222, I70223, I70228, I70229,I7092, I70231, I70232, I70233, I70234, I70235, I70238, I70239, I70241, I70242, I70243, I70244, I70245, I70248, I70249, I70261, I70262, I70263, I70268, I70269, I70291, I70292, I70293, I70298, I70299, I75011, I75012, I75013, I75019, I75021, I75022, I75023, I75029, I7025, I70209, I70301~3, I70308, I70309, I70311~3, I70318, I70319, I70321, I70322, I70323, I70328, I70329, I70331, I70332, I70333, I70334, I70335, I70338, I70339, I70341, I70342, I70343, I70344, I70345, I70348, I70349, I7035, I70361, I70362, I70363, I70368, I70369, I70391, I70392, I70393, I70398, I70399, I703601, I703602, I703603, I703608, I703609, I703611, I703612, I791, I798, I7389, I739, I743, I744, I96, I70261, I70262, I70263, I70268, I70269, E0852, E0952, E1152, E1352 |
| **Hypertension** | ICD-9 code:  401.X, 402.X, 403.X, 404.X, 405.X  ICD-10 code:  I10, I110, I120, I129, I130, I1311, I132, I150, I151, I152, I158, I159, I16X |
| **Diabetic nephropathy** | ICD-9 code:  250.4X, 585, 586, V42.0, V56.X, 403, 404  ICD-10 code:  E1121, E1122, E1129, E1321, E1322, E1329, E1165, N181, N182, N183, N184, N185, N186, N189, N19, Z940, Z4931, Z4901, Z4902, I129, I120, I1310, I130, I1311, I132 |
| **Diabetic retinopathy** | ICD-9 code:  250.5X  ICD-10 code:  E08311, E08319, E08321, E08329, E08331, E08339, E08341, E08349, E08351, E08359, E09311, E09319, E09321, E09329, E09331, E09339, E09341, E09349, E09351, E09359, E11311, E11319, E11321, E11329, E11331, E11339, E11341, E11349, E11351, E11359, E1139, E13311, E13319, E13321, E13329, E13331, E13339, E13341, E13349, E13351, E13359, E3139 |
| **Diabetic neuropathy** | ICD-9 code: 250.6X  ICD-10 code:  E0840, E0841, E0842, E0843, E0844, E0849, E0940, E0941, E0942, E0943, E0944, E0949, E1140, E1141, E1142, E1143, E1144, E1149, E1340, E1341, E1342, E1343, E1344, E1349 |
| **End stage renal disease (ESRD)** | ICD-9 Registry for catastrophic illness patients: 585  ICD-10 Registry for catastrophic illness patients: N185, N186, I120, I1311, I132 |
| **Cerebrovascular disease (CVA)** | ICD-9 code: 433.XX、434.XX、435.X、436、437.1、430、431、432.X  ICD-10 code: I638、I639、I63XX、I63XXX、I651、I658、I659、I65XX、I663、I668、I669、I66XX、I672、I6781、I6782、I6789、I679、I688、G450、G458、G451、G452、G459、I67841、I67848、I6789、I6789、I6781、I6782、I60X、I60XX、I61X、I62XX、I621、I629 |
| **Heart failure** | ICD-9 code: 428.X  ICD-10 code: I5020、I5021、I5022、I5023、I5030、I5031、I5032、I5033、I5040、I5041、I5042、I5043、I509、I501 |
| **Coronary heart disease (CHD)** | ICD-9 code: 410.XX、411.1、411.81、411.89、412、414.00-414.07、414.8、4149  ICD-10 code: I220、I221、I222、I228、I229、I230、I231、I232、I233、I234、I235、I236、I237、I238、I2101、I2102、I2109、I2111、I2119、I2121、I2129、I213、I214、I200、I240、I248、I249、I252、I2510、I25110、I25111、I25118、I25119、I255、I256、I25700、I25701、I25708、I25709、I25710、I25711、I25718、I25719、I25720、I25721、I25728、I25729、I25730、I25731、I25738、I25739、I25750、I25751、I25758、I25759、I25760、I25761、I25768、I25769、I25790、I25791、I25798、I25799、I25810、I25811、I25812、I2582、I2583、I2584、I2589、I255、I256、I2589、I259、I25 |
| **Medications code** | |
| **Metformin** | A034096100、A0340961G0、A034549100、A035324100、A0353241G0、A035665100、A0356651G0、A036201100、A0362011G0、A036204100、A0362041G0、A036348100、A036820100、A0368201G0、A036848100、A0368481G0、A038498100、A0384981G0、A038610100、A0386101G0、A038680100、A039546100、A040035100、A040047100、A0400471G0、A040098100、A040781100、A0407811G0、A0408351G0、A041233100、A0412331G0、A0417501G0、A042268100、A0422681G0、A042464100、A0424641G0、A042916100、A0429161G0、A044233100、A046686100、A048781100、A049531100、A049599100、A057265100、AB34096100、AB340961G0、AB35665100、AB356651G0、AB38610100、AB386101G0、AB40119100、AB408351G0、AB41233100、AB412331G0、AB49599100、AC34096100、AC340961G0、AC35665100、AC356651G0、AC36201100、AC362011G0、AC36204100、AC362041G0、AC36820100、AC368201G0、AC38680100、AC40047100、AC400471G0、AC40098100、AC40119100、AC401191G0、AC40781100、AC407811G0、AC407811G4、AC407811G7、AC408351G0、AC41233100、AC412331G0、AC417501G0、AC42916100、AC429161G0、AC44233100、AC487811G0、AC49531100、AC49599100、AC495991G0、AC57265100、AC57979100、AC58072100、AC580721G0、AC58257100、AC58534100、AC585341G0、AC59686100、AC596861G0、B018054100、B018160100、B024448100、  A046732100、A046733100、A057860100、A057861100、AB58071100、AC46733100、AC57799100、AC57860100、AC57861100、AC58071100、AC58954100、AC59300100、AC59393100、AC59759100、AC60134100、B023942100、B023943100、B023944100、B024005100、B024006100、B024459100、B024839100、B024876100、B025040100、B025041100、B025043100、B025453100、B025454100、B025455100、B025480100、B025481100、B025482100、B026109100、B026110100、BA24876100、BC24005100、BC24006100、BC24459100、BC24839100、BC24876100、BC25040100、BC25041100、BC25043100、BC25453100、BC25454100、BC25455100、BC25480100、BC25481100、BC25482100、BC25792100、BC25793100、BC25794100、BC26109100、BC26110100、BC26685100、BC26686100、BC27035100、BC27036100、BC27037100、BC27038100、BC27039100、BC27040100、BC27114100、BC27115100、BC27116100、BC27117100 |
| **Sulphonylurea** | A046074100、A046766100、AB46074100、AB46766100、AC46074100、AC46766100、AC57117100、AC58121100、AB58071100、AC58071100、AC58954100、AC59300100、AC59759100、AC60134100、B024876100、BA24876100、BC24876100、A021640100、A0216401G0、A034550100、A0345501G0、A034621100、A0346211G0、A035012100、A035670100、A035806100、A035813100、A035818100、A035886100、A035915100、A036068100、A036395100、A0363951G0、A036431100、A036862100、A036917100、A040233100、A0402331G0、A040304100、A040641100、A0406411G0、A041597100、A041805100、A050181100、AC216401G0、AC34550100、AC34621100、AC346211G0、AC35670100、AC35806100、AC36395100、AC363951G0、AC36431100、AC36862100、AC40233100、AC402331G0、AC41597100、AC41805100、AC50181100、AC501811G0、AC57921100、AC579211G0、B021143100、A029337100、A0293371G0、A029786100、A030512100、A030698100、A030970100、A034348100、A0343481G0、A034350100、A034736100、A0347361G0、A034893100、A035126100、A035414100、A0354141G0、A035518100、A035795100、A0357951G0、A036240100、A0362401G0、A036507100、A037445100、A038181100、A038181110、A038500100、A038681100、A039049100、A039144100、A040583100、A041563100、A042395100、A042888100、A042908100、A0429081G0、A043265100、A043520100、A0435201G0、A044172100、A044247100、A047070100、A048089100、AB30970100、AB34736100、AB347361G0、AB40583100、AB405831G0、AB42908100、AB429081G0、AB47070100、AB470701G0、AB48089100、AB57997100、AC29337100、AC293371G0、AC30698100、AC306981G0、AC30970100、AC309701G0、AC34348100、AC343481G0、AC34736100、AC34893100、AC35795100、AC357951G0、AC36240100、AC362401G0、AC38181100、AC38500100、AC39144100、AC39734100、AC397341G0、AC40583100、AC405831G0、AC41563100、AC42888100、AC42908100、AC429081G0、AC435201G0、AC44172100、AC441721G0、AC47070100、AC470701G0、AC48089100、AC480891G0、A037527100、A0375271G0、AC37527100、AC375271G0、B019621100、B019622100、B022564100、A046732100、A046733100、AC46733100、B024005100、B024006100、B026109100、B026110100、BC24005100、BC24006100、BC26109100、BC26110100、A036340100、A003792100、A029325100、A032831100、AC32831100、A006823100、A019070100、A024302100、A030305100、A0303051G0、A033501100、A040439100、AC30305100、AC303051G0、A033138100 |
| **Glinide** | A049061100、A049144100、AA49061100、AB49061100、AB57225100、AB57327100、AC49061100、AC57225100、AC57327100、AC58068100、B026269100、B023244100、B023245100、BC23244100、BC23245100、A052337100、AA52337100 |
| **Thiazolidinedione (TZD)** | A047130100、A048007100、A049560100、AA48007100、AB48007100、AC48007100、AC49560100、AC57872100、AC57897100、AC57799100、AC59393100、B024839100、BC24839100、BC26681100、BC26682100、BC26683100、A049615100、A049625100、AC49625100 |
| **𝛼 glucosidase inhibitor** | A047981100、A048898100、A049204100、A051205100、AB47981100、AB57312100、AC47981100、AC48898100、AC49204100、AC57312100、A051714100、AB51714100、AC51714100、AC58241100、AC58975100 |
| **Dipeptidyl peptidase-4 inhibitor**  **(DPP-4i)** | B025220100、B025453100、B025454100、B025455100、BC25220100、BC25453100、BC25454100、BC25455100、BC27467100、B025537100、BC25537100、BC25792100、BC25793100、BC25794100、BC27073100、BC27074100、BC26298100、BC26299100、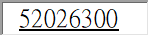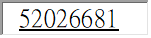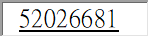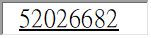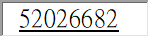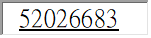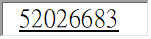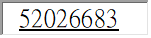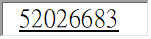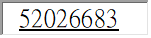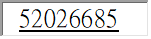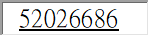BC26300100、BC26681100、BC26682100、BC26683100、BC26685100、BC26686100、AC59782100、B025306100、B025480100、B025482100、BC25306100、BC25480100、BC25481100、BC25482100、AC58620100、AC59308100、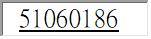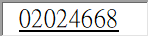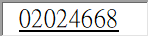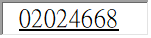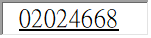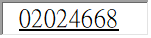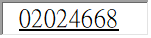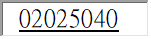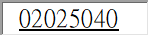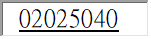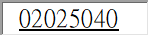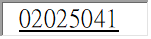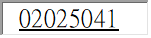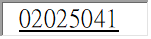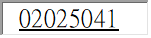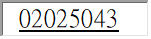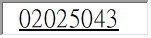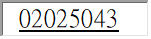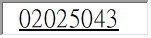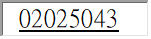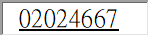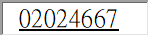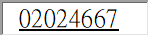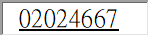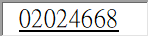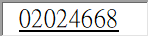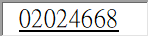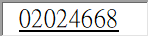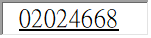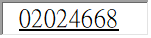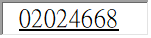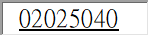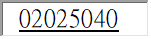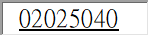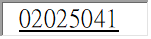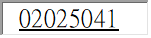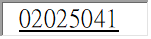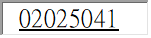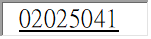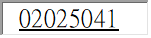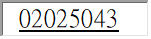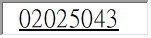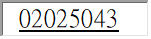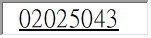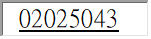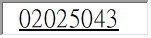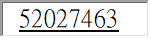AC60186100、B024668100、B025040100、B025041100、B025043100、BC24667100、BC24668100、BC25040100、BC25041100、BC25043100、BC27463100 |
| **Glucagon-like peptide-1 receptor agonist**  **(GLP-1 RA)** | K000914216、KC00914216、KC00978206、KC00979206、KC01080216、KC01107210、KC01107216 |
| **Sodium-Glucose Cotransporter-2 Inhibitors (SGLT2i)** | BC26405100、BC26406100、BC27035100、BC27036100、BC27037100、BC27038100、BC27039100、BC27040100、BC27073100、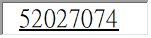BC27074100、BC26475100、BC26476100、BC27114100、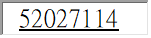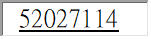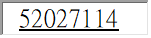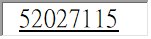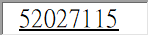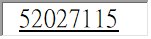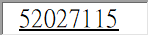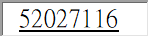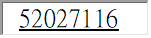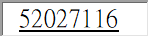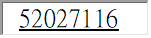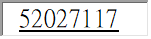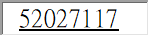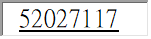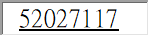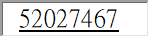BC27115100、BC27116100、BC27117100、BC27467100、BC26950100、BC27458100、BC27463100 |
| **Insulin** | A034983209、A034983299、A034984209、A034984299、B015613209、B015613299、B015635209、B015635299、B015735209、B015735299、B015801209、B015801299、B015806209、B015806299、B017189209、B017189299、B018823209、B018823299、B018824209、B018824261、B018824299、B018826209、B018826299、B018827209、B018827299、B018828209、B018828299、B018830209、B018830299、B018831209、B018831261、B018831299、B018832209、B018832261、B018832299、B018836209、B018836299、B018837209、B018837299、B018993209、B018993299、B018994209、B018994299、B019085209、B019085299、B019172209、B019172299、B019174209、B019174299、B019469209、B019469299、B019568209、B019568299、B019650209、B019650261、B019650266、B019650299、B019651261、B019657209、B019657299、B019686209、B019686299、B019687209、B019687261、B019687266、B019687299、B019865209、B019865299、B019896209、B019896299、B020306261、B020306266、B020306299、B020307261、B020307266、B020307299、B020308261、B020308266、B020308299、B020309261、B020309266、B020309299、B020310261、B020310266、B020310299、B020311261、B020311266、B020311299、B020324209、B020324299、B020325209、B020325299、B020678209、B020678299、B021155209、B021155299、B021326209、B021326299、B021410261、B021411261、B021411266、B021412261、B021412266、B021413261、B021413266、B021413299、B021420261、B021420266、J000110209、J000110299、J000111209、J000111299、K000595266、K000596266、K000652266、K000653209、K000653299、K000656266、K000657209、K000657299、K000658209、K000658299、K000659209、K000659299、K000660266、K000661266、K000663209、K000663299、K000682209、K000682299、K000685266、K000685299、K000697266、K000728266、K000729266、K000730209、K000738266、K000738299、K000739209、K000739266、K000739299、K000741209、K000741299、K000742209、K000742299、K000745209、K000750209、K000750299、K000760209、K000760299、K000768266、K000795266、K000803266、K000810266、K000820266、K000823266、K000898266、K000899266、K000900266、K000908266、K000909266、KC00595266、KC00596266、KC00653209、KC00657209、KC00663209、KC00682209、KC00697266、KC00728266、KC00729266、KC00739209、KC00745209、KC00760209、KC00768266、KC00795266、KC00803266、KC00810266、KC00820266、KC00823266、KC00898266、KC00899266、KC00900266、KC00908266、KC00909266、KC00986266、KC01011272、KC01054266、KC01080216、KC01053216 |
| **Statins** | A044998100、A046402100、A047348100、A047775100、A047907100、A047924100、A047928100、A048241100、A048562100、A048608100、A048813100、A048879100、A048926100、A049190100、A049226100、A049288100、A049360100、A049535100、A049543100、A049661100、A049672100、A049699100、A049792100、A049841100、A049997100、A050086100、A051598100、A051732100、A052301100、A052465100、A052479100、A054967100、A055583100、A055895100、A055956100、A055967100、A056319100、A056629100、A056739100、A056791100、A057176100、A057194100、A057216100、A057372100、A057809100、A057930100、A058207100、AA48879100、AA49226100、AA49288100、AA49543100、AA51598100、AA52530100、AA55268100、AA55272100、AA56739100、AA57372100、AA57774100、AA57802100、AA57805100、AA57843100、AA57880100、AA57930100、  AA57950100、AA58282100、AA58648100、AB47348100、AB48879100、AB49226100、AB49288100、AB49543100、AB51732100、AB52530100、AB54967100、AB55268100、AB55272100、AB57133100、AB57194100、AB57267100、AB57772100、AB57805100、AB57843100、AB57930100、AB57940100、AB57967100、AB58049100、AC44998100、AC46402100、AC47348100、AC47775100、AC47907100、AC47924100、AC47928100、AC48608100、AC48813100、AC48879100、AC48926100、AC49190100、AC49226100、AC49288100、AC49360100、AC49535100、AC49543100、AC49661100、AC49672100、AC49699100、AC49792100、AC49841100、AC49997100、AC50086100、AC51598100、AC51732100、AC52301100、AC52465100、AC52479100、AC52530100、AC54967100、AC55268100、AC55272100、AC55583100、AC55895100、AC55952100、AC55956100、AC56319100、AC56629100、AC56682100、AC56739100、AC56791100、AC56804100、AC56806100、AC57130100、AC57133100、AC57176100、AC57194100、AC57216100、AC57267100、AC57772100、AC57774100、AC57802100、AC57803100、AC57805100、AC57806100、AC57809100、AC57843100、AC57880100、AC57930100、AC57940100、AC57950100、AC57967100、AC58041100、AC58049100、AC58067100、AC58078100、AC58098100、AC58207100、AC58211100、AC58270100、AC58282100、AC58291100、AC58315100、AC58316100、AC58366100、AC58384100、AC58396100、AC58401100、AC58411100、AC58525100、AC58526100、AC58579100、AC58605100、AC58621100、AC58622100、AC58633100、AC58639100、AC58648100、AC58813100、AC58822100、AC59192100、AC59193100、AC59240100、AC59251100、AC59265100、AC59266100、AC59398100、AC59649100、AC59652100、AC59887100、AC60114100、AC60175100、B020037100、B021023100、B021198100、B021199100、B022886100、B022889100、B022890100、B023144100、B023506100、B023556100、B023970100、B024129100、B024131100、B024250100、B024252100、B024339100、B024391100、B024392100、B024534100、B024535100、B024597100、B024868100、B025200100、B025201100、B025211100、B025337100、B025350100、B025810100、B026132100、B026147100、B026226100、BA25200100、BA25201100、BA25337100、BA25797100、BA25798100、BA26332100、BA26504100、BB25200100、BB25201100、BB25337100、BC20037100、BC21198100、BC21199100、BC22886100、BC22889100、BC22890100、BC23506100、BC23556100、BC23970100、BC24129100、BC24131100、BC24250100、BC24252100、BC24339100、BC24391100、BC24392100、BC24597100、BC24868100、BC25211100、BC25350100、BC25796100、BC25797100、BC25798100、BC25810100、BC26028100、BC26132100、BC26147100、BC26226100、BC26332100、BC26350100、BC26367100、BC26368100、BC26401100、BC26426100、BC26427100、BC26497100、BC26504100、BC26505100、BC26543100、BC26544100、BC26582100、BC26643100、BC26857100、BC26900100、BC27002100、BC27044100、BC27256100、BC27283100、BC27339100 |
| **Ezetiminde** | PFC018M、PFC066M、PFC081M、PFC084M |
| **Anti-platelet drugs** | PGC110M、PLC044M、PLC044M、PTA056M、P4A094M、P4A094M、PLC031M、PLC041M、PLC056M、PZA018M、PZA252M、PLC051M、PLC054M、PFE007M、PFE008M |
| Thyroxine | PMG027M、PMG026M、PMG018M、P2A091M、P2A093M |
| Anti-thyroid drugs:  Methimazole  Carbimazole  Propylthiouracil | PMG008M  PMG004M  PMG024M |
| **Definition of outcomes** | |
| MACE refers to non-fetal myocardial infarction, stroke, and heart failure leading to hospital admission. Any one of the three diagnoses were **identified using ICD-9 and ICD-10 codes based on the top five coding for any admission or on the top three coding for more than twice outpatient visits or MACE procedure codes/thrombolysis therapy in inpatient or outpatient setting. The index date was defined as the first date comfort to the MACE definition.** | |
| **Non-fetal myocardiol infarction (A.+B.+C.)** | 1. ICD9 code : 410.x   ICD10 code :I21,I22,I25   1. procedural codes used by the Taiwan NHI including percutaneous coronary intervention (33076A, 33076B, 33077A, 33077B, 33078A, 33078B), coronary artery bypass surgery (68023A, 68023B, 68024A, 68024B, 68025A, 68025B), and (67030A, 67030B,68006A, 68006B, 69004A, 69004B) 2. thrombolysis therapy (B016526248, K000743248, K000744238, KC00743248). |
| **Stroke** | ICD9 code : 430.x–437.x  ICD10 code : I60,I61,I62,I63 |
| **Heart failure for admission** | Inpatient disease codes  ICD9 code : 428.x  ICD10 code : I5020, I5021, I5022, I5023, I5030, I5031, I5032, I5033, I5040, I5041, I5042, I5043 I509, I501 |
